# Incidence of and risk factors for newly diagnosed hyperkalemia after hospital discharge in non-dialysis-dependent CKD patients treated with RAS inhibitors

**DOI:** 10.1371/journal.pone.0184402

**Published:** 2017-09-06

**Authors:** Yuki Saito, Hiroyuki Yamamoto, Hideki Nakajima, Osamu Takahashi, Yasuhiro Komatsu

**Affiliations:** 1 Division of Nephrology, Department of Internal Medicine, St Luke’s International Hospital, Tokyo, Japan; 2 Division of Internal Medicine, Keio University Hospital, Tokyo, Japan; 3 Department of Healthcare Quality Assessment, Graduate School of Medicine, The University of Tokyo, Tokyo, Japan; 4 Information System Center, St Luke’s International Hospital, Tokyo, Japan; 5 Division of General Internal Medicine, Department of Internal Medicine, St Luke’s International Hospital, Tokyo, Japan; 6 Division of Clinical Epidemiology, Graduate School of Public Health, St Luke’s International University, Tokyo, Japan; Kaohsiung Medical University Hospital, TAIWAN

## Abstract

**Introduction:**

Renin-angiotensin system (RAS) inhibitors have been increasingly prescribed due to their beneficial effects on end-organ protection. Iatrogenic hyperkalemia is a well-known life-threatening complication of RAS inhibitor use in chronic kidney disease (CKD) patients. We hypothesized that CKD patients treated with RAS inhibitors frequently develop hyperkalemia after hospital discharge even if they were normokalemic during their hospitalization because their lifestyles change substantially after discharge. The present study aimed to examine the incidence of newly diagnosed hyperkalemia, the timing of hyperkalemia, and its risk factors in CKD patients treated with RAS inhibitors at the time of hospital discharge.

**Methods:**

We retrospectively enrolled patients aged 20 years or older with CKD G3-5 (estimated glomerular filtration rate < 60 mL/min/1.73 m^2^) and who were treated with RAS inhibitors and discharged from St. Luke’s International Hospital between July 2011 and December 2015. Patients who were under maintenance dialysis or had hyperkalemic events before discharge were excluded. Data regarding the patients’ age, sex, CKD stage, diabetes mellitus status, malignancy status, combined use of RAS inhibitors, concurrent medication, and hyperkalemic events after discharge were extracted from the hospital database. Our primary outcome was hyperkalemia, defined as serum potassium ≥ 5.5 mEq/L. Multiple logistic regression and Kaplan-Meier analyses were performed to identify the risk factors for and the timing of hyperkalemia, respectively.

**Results:**

Among the 986 patients, 121 (12.3%) developed hyperkalemia after discharge. In the regression analysis, relative to CKD G3a, G3b [odds ratio (OR): 1.88, 95% confidence interval 1.20–2.97] and G4-5 (OR: 3.40, 1.99–5.81) were significantly associated with hyperkalemia. The use of RAS inhibitor combinations (OR: 1.92, 1.19–3.10), malignancy status (OR: 2.10, 1.14–3.86), and baseline serum potassium (OR: 1.91, 1.23–2.97) were also significantly associated with hyperkalemia. The Kaplan-Meier analysis showed that hyperkalemia was most frequent during the early period after discharge, particularly within one month.

**Conclusion:**

Hyperkalemia was frequent during the early period after discharge among previously normokalemic CKD patients who were treated with RAS inhibitors. Appropriate follow-up after discharge should be required for these patients, particularly those with advanced CKD or malignancy status, such as hematological malignancy or late-stage malignancy, and those who are treated with multiple RAS inhibitors.

## Introduction

Renin-angiotensin system inhibitors (RAS inhibitors) are frequently prescribed because of their beneficial effects on cardiovascular event reduction[1][2] and end-organ protection[3], including renoprotection[4][5]. Angiotensin-converting enzyme (ACE) inhibitors and angiotensin-receptor blockers (ARBs), which are both RAS inhibitors, are commonly used to treat hypertension, and nephrologists and cardiologists are not the only physicians prescribing RAS inhibitors. Spironolactone, which is another type of RAS inhibitor, is also widely used for the reduction of mortality and morbidity in heart failure patients[6]. Despite these beneficial effects, RAS inhibitors also have a severe, life-threatening adverse effect, hyperkalemia[7][8]. Accumulating evidence suggests that the incidence of RAS inhibitor-induced hyperkalemia is increasing[9].

However, little is known regarding the incidence of and risk factors for hyperkalemia in chronic kidney disease (CKD) patients who are treated with RAS inhibitors. The National Kidney Foundation Kidney Disease Outcomes Quality Initiative (NKF KDOQI) guidelines recommend reducing serum potassium concentrations and educating patients to avoid high-potassium diets after the initiation of or a change in the dose of an ACE inhibitor or ARB[10]. Specifically, lifestyle modification is required to avoid hyperkalemia in patients treated with RAS inhibitors.

However, few studies have focused on the impact of lifestyle modifications on serum potassium concentrations. We focused on hospital discharge because previous studies of early hospital readmission suggest that post-discharge environments affect patients’ health status[11][12]. We hypothesized that even when the serum concentration is within the normal range before or during hospitalization, CKD patients who are treated with RAS inhibitors frequently develop hyperkalemia after hospital discharge because their lifestyle changes substantially after they leave the hospital.

Therefore, the present study aimed to examine the incidence of newly diagnosed hyperkalemia, the timing of hyperkalemia, and its risk factors in non-dialysis-dependent CKD patients treated with RAS inhibitors after hospital discharge.

## Methods

### Study design

This study was a single-center retrospective cohort study performed at a teaching hospital (St Luke’s International Hospital, Tokyo, Japan). Patients aged 20 years or older with CKD G3-5 who were treated with a RAS inhibitor after hospital discharge between July 2011 and December 2015 were investigated. We excluded patients who underwent maintenance dialysis (both hemodialysis and peritoneal dialysis) and those who progressed to hyperkalemia within 120 days prior to discharge. In particular, we focused on patients who were newly diagnosed with hyperkalemia. If a patient was hospitalized several times during the study period, we only included the initial hospitalization, and the other hospitalizations were excluded. Dietary education was provided for these patients during the admission period as appropriate. All aspects of this study were approved by the Institutional Review Board of St Luke’s International Hospital Ethics Committee (approval number 16-J003). Informed consent was waived because of the retrospective nature of the study.

### Data collection

All data were extracted from the database of St Luke’s International Hospital, Japan. Data regarding age, sex, CKD stage, combined use of RAS inhibitors, diabetes mellitus status, malignancy status, and the use of concomitant drugs, such as nonsteroidal anti-inflammatory drugs (NSAIDs), β-blockers, potassium salt substitutes, potassium binders (sodium polystyrene sulfonate and calcium polystyrene sulfonate), and diuretics (loop diuretics and/or thiazide diuretics), were investigated.

### Definitions

RAS inhibitors included the following four categories: ACE inhibitors, ARBs, aldosterone antagonists, and direct renin inhibitors. The use of combination RAS inhibitors was defined as the use of more than two categories of RAS inhibitor. If a patient was treated with two medications in the same category, the treatment was not considered combination use.

In this study, CKD stage G3-5 was defined as an estimated glomerular filtration rate (eGFR) < 60 mL/min/1.73 m^2^ in a blood test performed immediately prior to hospital discharge. The eGFR was estimated using the equation proposed by the Japanese Society of Nephrology[13]. Hyperkalemia was defined as serum potassium ≥ 5.5 mEq/L using previously defined thresholds for hyperkalemia[14][15][16], and newly diagnosed hyperkalemia was defined as hyperkalemia diagnosed between 1 and 120 days after discharge[17]. For malignancy, we included patients receiving treatment (including chemotherapy, radiotherapy, hormone therapy, surgery, and other) or in palliative care.

### Statistical analysis

All statistical analyses were conducted using STATA software version 12 (STATA Corp., TX, USA). Continuous variables are presented as the means (standard deviation), and categorical variables are presented as counts (percentage). We constructed a multiple logistic regression model to evaluate the association between hyperkalemia and its possible risk factors. The inclusion of variables in the models was based on existing knowledge of hyperkalemia in CKD patients. All continuous variables were categorized as appropriate. To calculate the timing of hyperkalemia development, a Kaplan-Meier curve and a smoothed hazard estimate curve were drawn. A two-sided p-value < 0.05 was considered statistically significant.

## Results

### Baseline characteristics of subjects

**Fig 1:**
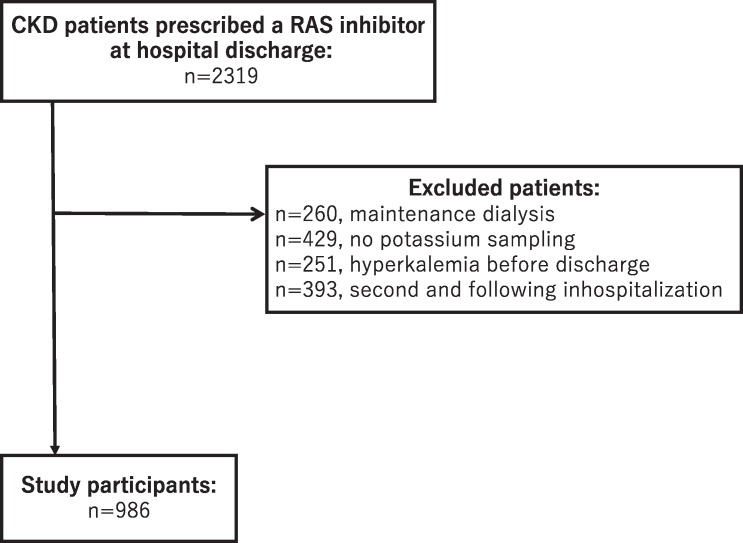
Method of patient selection. CKD, chronic kidney disease; RAS, renin-angiotensin system.

In total, 2,319 CKD patients were prescribed a RAS inhibitor during our observation period. Overall, 1,333 patients were excluded, and the final sample included 986 patients (Fig 1). A total of 777 patients (78.8%) completed the follow-up period. Table 1 shows the baseline characteristics of the patients. The mean age of our study participants was 76.0 years (SD, 11.3). The mean eGFR was 45.4 mL/min/1.73 m^2^ (SD, 11.7), and 88.1% of the participants were CKD G3. The top three departments from which the patients were discharged were cardiology (n = 382, 38.7%), cardiac surgery (n = 110, 11.2%), and gastroenterology (n = 76, 7.7%). In the entire population, 686 patients (69.6%) underwent transthoracic echocardiogram, and 100 of them (14.6%) had an ejection fraction (EF) less than or equal to 35%. Additionally, 17.8% of the participants (n = 176) were using more than two types of RAS inhibitors. Details of the RAS inhibitor combinations are listed in S1 Table. Of the 986 patients, 121 patients (12.3%) developed new hyperkalemia after discharge. Of those patients who had been reliably prescribed the same dose of RAS inhibitor before admission (n = 597, 60.5%), 10.7% developed new hyperkalemia.

**Table 1:** Baseline characteristics of the patients.

Variable	All, n = 986	Hyperkalemia, n = 121	No hyperkalemia, n = 865	*p* values
**Male, n (%)**	574 (58.2)	72 (59.5)	502 (58.0)	0.84
**Age, mean (SD), year**	76.0 (11.3)	76.4 (10.7)	76.0 (11.4)	0.71
**20–64 years, n (%)**	144 (14.6)	19 (15.7)	125 (14.5)	0.89
**65–79 years, n (%)**	428 (43.4)	53 (43.8)	375 (43.4)	
**≥80 years, n (%)**	414 (42.0)	49 (40.5)	365 (42.2)	
**eGFR, mean (SD), mL/min/1.73 m**^**2**^	45.4 (11.7)	40.5 (13.7)	46.1 (11.3)	<0.01
**CKD stage**				<0.01
**CKD G3a, n (%)**	604 (61.3)	51 (42.1)	553 (63.9)	
**CKD G3b, n (%)**	265 (26.9)	41 (33.9)	224 (25.9)	
**CKD G4-G5, n (%)**	117 (11.9)	29 (24.0)	88 (10.2)	
**Baseline serum K, mean (SD), mEq/L**	4.26 (0.47)	4.35 (0.51)	4.24 (0.46)	0.03
**RAS inhibitor type**				
**ACE inhibitor/ARB, n (%)**	889 (90.2)	106 (87.6)	783 (90.5)	0.33
**Aldosterone antagonist, n (%)**	250 (25.4)	47 (38.8)	203 (23.5)	<0.01
**Direct renin inhibitor, n (%)**	13 (1.3)	2 (1.7)	11 (1.3)	0.67
**Combined use of RAS inhibitors, n (%)**	176 (17.8)	38 (31.4)	138 (16.0)	<0.01
**Diabetes mellitus, n (%)**	501 (50.8)	63 (52.1)	438 (50.6)	0.77
**Malignancy, n (%)**	109 (11.1)	18 (14.9)	91 (10.5)	0.16
**Concomitant drugs**				
**NSAIDs, n (%)**	59 (6.0)	6 (5.0)	53 (6.1)	0.84
**β-blockers, n (%)**	464 (47.1)	63 (52.1)	401 (46.4)	0.25
**Potassium binders, n (%)**	20 (2.0)	3 (2.5)	17 (2.0)	0.73
**Potassium salt substitutes, n (%)**	47 (4.8)	5 (4.1)	42 (4.9)	1.00
**Loop and/or thiazide diuretics, n (%)**	405 (41.1)	70 (57.9)	335 (38.7)	<0.01

CKD, chronic kidney disease; eGFR, estimated glomerular filtration rate; RAS, renin-angiotensin system; ACE, angiotensin-converting enzyme; ARB, angiotensin-receptor blockers; NSAIDs, nonsteroidal anti-inflammatory drugs.

### Risk factor analysis

Table 2 shows the results of a logistic regression analysis to clarify the risk factors of the newly diagnosed hyperkalemia. In the multivariable logistic regression (model 1), relative to CKD G3a, CKD G3b (odds ratio (OR) 1.88, 95% confidence interval (CI) [1.20–2.97]) and G4+5 (OR 3.40 [1.99–5.81]) showed a dose-dependent association with hyperkalemia. Combination RAS inhibitor use, malignancy, and baseline serum potassium were also significantly associated with hyperkalemia. The ORs were 1.92 (1.19–3.10), 2.10 (1.14–3.86), and 1.91 (1.23–2.97), respectively. Additionally, the usage of loop and/or thiazide diuretics was significantly associated with hyperkalemia in model 1. When we added the variable, EF≤35% (model 2), these results were not altered except for a diminished significance of the usage of loop and/or thiazide diuretics, which was due to confounding.

**Table 2:** Risk factors for a new diagnosis of hyperkalemia.

	Univariable	Multivariable	Multivariable
	N = 986	model 1, N = 986	model 2, N = 686
**Male**	1.06 [0.72–1.57]	1.11 [0.73–1.70]	1.38 [0.84–2.27]
**Age**			
**20–64 years**	Ref.	Ref.	Ref.
**65–79 years**	0.93 [0.53–1.63]	0.82 [0.45–1.48]	0.74 [0.37–1.46]
**≥80 years**	0.88 [0.50–1.56]	0.78 [0.42–1.44]	0.81 [0.40–1.63]
**CKD stage**			
**CKD G3a**	Ref.	Ref.	Ref.
**CKD G3b**	1.98 [1.28–3.08]	1.88 [1.20–2.97]	2.24 [1.33–3.77]
**CKD G4-5**	3.57 [2.15–5.94]	3.40 [1.99–5.81]	3.73 [2.00–6.94]
**Diabetes mellitus**	1.06 [0.72–1.55]	1.01 [0.67–1.53]	1.22 [0.76–1.97]
**Combined use of RAS inhibitors**	2.41 [1.58–3.69]	1.92 [1.19–3.10]	1.85 [1.08–3.19]
**Malignancy**	1.49 [0.86–2.57]	2.10 [1.14–3.86]	2.60 [1.17–5.81]
**Concomitant drugs**			
**NSAIDs**	0.80 [0.34–1.90]	0.83 [0.33–2.08]	0.69 [0.20–2.41]
**β-blockers**	1.26 [0.86–1.84]	1.09 [0.71–1.67]	1.11 [0.67–1.82]
**Potassium binders**	1.27 [0.37–4.39]	1.01 [0.28–3.65]	1.21 [0.31–4.69]
**Potassium salt substitutes**	0.84 [0.33–2.18]	1.02 [0.38–2.73]	0.99 [0.36–2.70]
**Loop and/or thiazide diuretics**	2.17 [1.48–3.19]	1.80 [1.16–2.79]	1.46 [0.88–2.42]
**Baseline serum K (unit: 1.0 mEq/L)**	1.66 [1.09–2.52]	1.91 [1.23–2.97]	1.67 [1.02–2.74]
**EF≤35%**	1.13 [0.62–2.06] ^a^		0.99 [0.51–1.91]

CKD, chronic kidney disease; RAS, renin-angiotensin system; NSAIDs, nonsteroidal anti-inflammatory drugs; Ref, Reference; EF, ejection fraction. Each cell shows the point estimates of the odds ratios and 95% confidence intervals.

^a^ Result of N = 686

### Types and stages of malignancy

We then compared the incidence of hyperkalemia between different types and stages of malignancy (S2 Table). The incidence of hyperkalemia tended to be higher than that of carcinoma in patients with hematological malignancies. Similarly, late-stage malignancy patients tended to have a higher incidence of hyperkalemia than early-stage patients.

### Timing of hyperkalemia development

**Fig 2:**
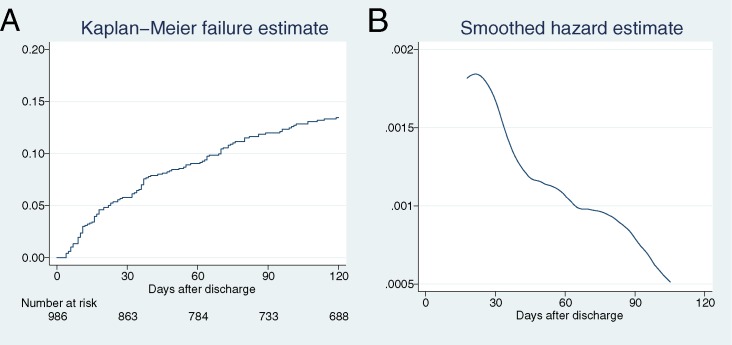
Cumulative incidence of hyperkalemia for the entire cohort. Kaplan-Meier curve; (B) Smoothed hazard estimate.

We then evaluated the timing of newly diagnosed hyperkalemia after discharge. Fig 2 shows the Kaplan-Meier curve and the smoothed hazard estimate. Fifty-five patients (45.5% of the hyperkalemic patients) developed hyperkalemia within 30 days. We subsequently re-performed the same analysis for patients who had undergone potassium sampling at least once within 0–30, 31–60, 61–90, and 91–120 days after discharge, respectively (S1 Fig). In total, 315 patients had complete potassium samples. A total of 56 patients (17.8%) developed hyperkalemia within 120 days after discharge, and 23 patients (7.3% of all sampled patients, 41.1% of the hyperkalemia patients) developed hyperkalemia within 30 days after discharge.

## Discussion

In this study, we investigated the incidence and risk factors of newly diagnosed hyperkalemia after hospital discharge in non-dialysis-dependent CKD patients who were treated with RAS inhibitors. The results showed that new diagnoses of hyperkalemia occurred in 10.7–12.3% of the CKD patients after the prescription of RAS inhibitors. We identified advanced CKD stage, the use of RAS inhibitor combinations, malignancy, and baseline serum potassium level as risk factors. Based on the Kaplan-Meier analysis, we found that hyperkalemia occurred most frequently during the early period after the discharge, i.e., within a month.

To the best of our knowledge, this study provides the first demonstration of the incidence of newly diagnosed hyperkalemia after hospital discharge in CKD patients treated with RAS inhibitors. Although we excluded patients who developed hyperkalemia before discharge, 12.3% of the study participants developed new hyperkalemia. In addition, when we limited the analysis to patients who had been prescribed the same dose of RAS inhibitor used prior to their admission, 10.7% of the patients developed new hyperkalemia. A recent study showed that hyperkalemia occurs in 2.6% of emergency department visits and 3.5% of hospital admissions[18]. Compared to these previous studies, our study population had a high incidence of hyperkalemia. Additionally, our study clearly showed that CKD patients treated with RAS inhibitors frequently develop hyperkalemia after hospital discharge, even if they were normokalemic before discharge. Normokalemia during hospitalization does not guarantee normokalemia after discharge. Hospitalized patients usually consume an appropriate amount of potassium, and physicians frequently monitor the serum potassium concentration. However, once a patient is discharged from the hospital, potassium intake is not under the physician’s control. Dramatic lifestyle alterations, such as medication changes, volume depletion, or an increase in potassium intake, might lead to hyperkalemia after discharge. Patient education and appropriate serum potassium monitoring after discharge should be required for all CKD patients treated with RAS inhibitors, regardless of their serum potassium concentration during hospitalization.

In this analysis, we identified advanced CKD stage, the use of RAS inhibitor combinations, malignancy, and high baseline serum potassium levels as risk factors for new hyperkalemia diagnosis. CKD progression[14] and the use of RAS inhibitor combinations[19] were previously reported to be risk factors for hyperkalemia, which supports the validity of our study. In contrast, the use of NSAIDs[20], β-blockers[21], and potassium salt substitutes[22], which are known risk factors for hyperkalemia, were not associated with hyperkalemia in our study. The number of patients using these medications was limited, and this study might have lacked sufficient power for the detection of these risk factors.

This pioneering work shows the relationship between malignancy and hyperkalemia. Patients with hematological malignancies or late-stage malignancies had a higher incidence of hyperkalemia. Our study indicates that physicians must reconsider prescribing RAS inhibitors to CKD patients with malignancies. The discontinuation or dose adjustment of RAS inhibitors might be a choice for these patients.

Tumor lysis syndrome (TLS) is known to cause hyperkalemia in cancer patients[23], but no patients in our study met the TLS criteria. Patients with active TLS are usually hospitalized and are unlikely to be discharged until TLS becomes inactive. Patients with malignancy tend to progress to cachexia[24]; thus, the eGFR might be overestimated, which could explain the common incidence of hyperkalemia in malignancy patients. In addition, because skeletal muscles serve as a reservoir for potassium[25], cancer cachexia patients may have a lower capacity to buffer increases in the extracellular potassium concentrations. Furthermore, because acute kidney injury (AKI) is common in cancer patients[26], these patients may have developed AKI-associated hyperkalemia. Further studies are required to clarify the mechanism underlying the common incidence of hyperkalemia in malignancy patients.

According to the Kaplan-Meier curve (Fig 2), hyperkalemia was most frequent during the early days after hospital discharge. As shown in Fig 2, 45.5% of the hyperkalemia patients were diagnosed within 30 days after discharge. Our result indicates that early follow-up after discharge is necessary for CKD patients treated with RAS inhibitors.

The strength of our study is that it included all CKD patients who were admitted to our teaching hospital. In our study, CKD G3 patients composed 88.1% of the study participants, which is consistent with the finding that CKD G3 patients make up the largest population of all CKD patients[27]. Compared to a previous study that was limited to moderate to severe CKD patients who were followed in a low-clearance clinic[14], our study population might be more representative of real world CKD patients.

Several limitations of our study merit emphasis. First, our study subjects were limited to patients for whom potassium samples were available. We may have missed patients who transferred to different hospitals or had no follow-up visits after discharge. Additionally, we could not determine hyperkalemic events experienced by the study population before the study period. The present study is based on a single-center study, which might limit the generalizability of the conclusions and the completeness of information available in electronic health records. Second, hyperkalemia is usually asymptomatic; thus, the incidence of hyperkalemia can be affected by the frequency of laboratory tests. Third, we defined hyperkalemia as potassium ≥ 5.5 mEq/L, and we did not limit it to severe cases. Fourth, we lack data on potassium intake during or after hospitalization. Potassium intake varies between cultures and countries[28], and Japanese potassium consumption is lower than that of Western countries[29].

## Conclusion

We demonstrated that hyperkalemia frequently occurs after hospital discharge in previously normokalemic CKD patients treated with RAS inhibitors. Advanced CKD, the use of RAS inhibitor combinations, malignancy status, and high baseline serum potassium levels were identified as risk factors for hyperkalemia. Appropriate follow-up should be required for these populations within the early days after discharge.

## Supporting information

S1 Table Details of the RAS inhibitor combinations. ACE, angiotensin-converting enzyme; ARB, angiotensin-receptor blockers.
(PDF)

S2 Table Incidence of newly diagnosed hyperkalemia by the types and stages of the malignancies. *: For malignancy stage, we reviewed all images and pathologies within 180 days before discharge, and patients were clinically or pathologically staged according to the 7^th^ edition of the UICC TNM Staging System. Stages 1/2 were defined as early stage, and stages 3/4 were defined as late stage.
†: Fisher’s exact test
(PDF)

S1 Fig Cumulative incidence of hyperkalemia in the 315 patients. (A) Kaplan-Meier curve; (B) Smoothed hazard estimate
(PDF)
