# Supplementary material for: Incidence of and risk factors for newly diagnosed hyperkalemia after hospital discharge in non-dialysis-dependent CKD patients treated with RAS inhibitors
Source: PLoS One. 2017 Sep 6;12(9):e0184402. doi: 10.1371/journal.pone.0184402 (PMC5587314; doi:10.1371/journal.pone.0184402)
Supplement: S1 Table — ACE, angiotensin-converting enzyme; ARB, angiotensin-receptor blockers. (PDF) [file pone.0184402.s001.pdf]

S1 Table. Details of the RAS inhibitor combinations

Combinations of RAS inhibitors	Results
**3 drugs**	
ACE inhibitor + Aldosterone antagonist + Direct renin inhibitor, n (%)	1 (0.6)
ARB + Aldosterone antagonist + Direct renin inhibitor, n (%)	1 (0.6)
**2 drugs**	
ACE inhibitor + ARB, n (%)	12 (6.8)
ARB + Direct renin inhibitor, n (%)	6 (3.4)
ARB + Aldosterone antagonist, n (%)	77 (43.8)
ACE inhibitor + Aldosterone antagonist, n (%)	79 (44.9)

ACE, angiotensin-converting enzyme; ARB, angiotensin-receptor blockers.
