# Supplementary material for: Incidence of and risk factors for newly diagnosed hyperkalemia after hospital discharge in non-dialysis-dependent CKD patients treated with RAS inhibitors
Source: PLoS One. 2017 Sep 6;12(9):e0184402. doi: 10.1371/journal.pone.0184402 (PMC5587314; doi:10.1371/journal.pone.0184402)
Supplement: S2 Table — *: For malignancy stage, we reviewed all images and pathologies within 180 days before discharge, and patients were clinically or pathologically staged according to the 7th edition of the UICC TNM Staging System. Stages 1/2 were defined as early stage, and stages 3/4 were defined as late stage. †: Fisher’s exact test (PDF) [file pone.0184402.s002.pdf]

S2 Table. Incidence of newly diagnosed hyperkalemia by the types and stages of the malignancies

	**Patients, n**	**Newly diagnosed hyperkalemia cases, n (%)**	***p* value†**
**Types**			0.21
**Carcinoma**	89	13 (14.6)	
**Hematological malignancies**	17	5 (29.4)	
**Sarcoma**	3	0 (0)	
**Stages***			0.19
**Early-stage**	31	2 (6.5)	
**Late-stage**	48	10 (20.8)	
**Unknown, unclassifiable**	30	6 (20.0)	

*: For malignancy stage, we reviewed all images and pathologies within 180 days before discharge, and patients were clinically or pathologically staged according to the 7th edition of the UICC TNM Staging System. Stages 1/2 were defined as early stage, and stages 3/4 were defined as late stage.

†: Fisher's exact test
