# Supplementary figures and images for: Incidence of and risk factors for newly diagnosed hyperkalemia after hospital discharge in non-dialysis-dependent CKD patients treated with RAS inhibitors
Source: PLoS One. 2017 Sep 6;12(9):e0184402. doi: 10.1371/journal.pone.0184402 (PMC5587314; doi:10.1371/journal.pone.0184402)

**A**

Kaplan-Meier failure estimate

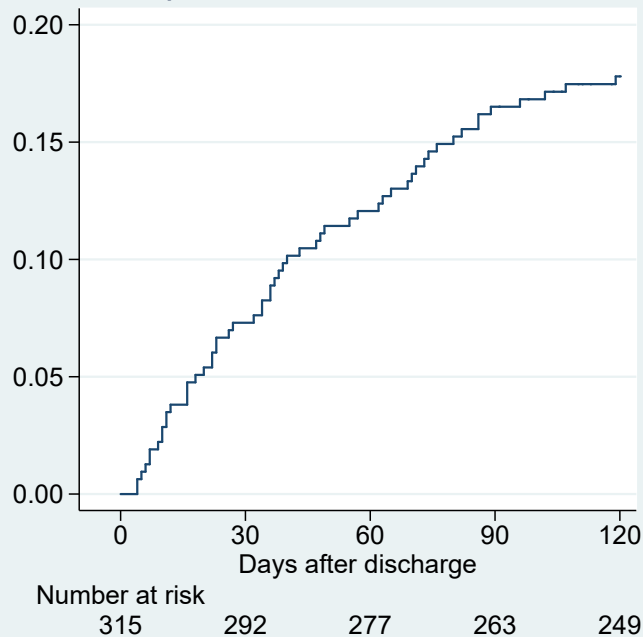**B**

Smoothed hazard estimate

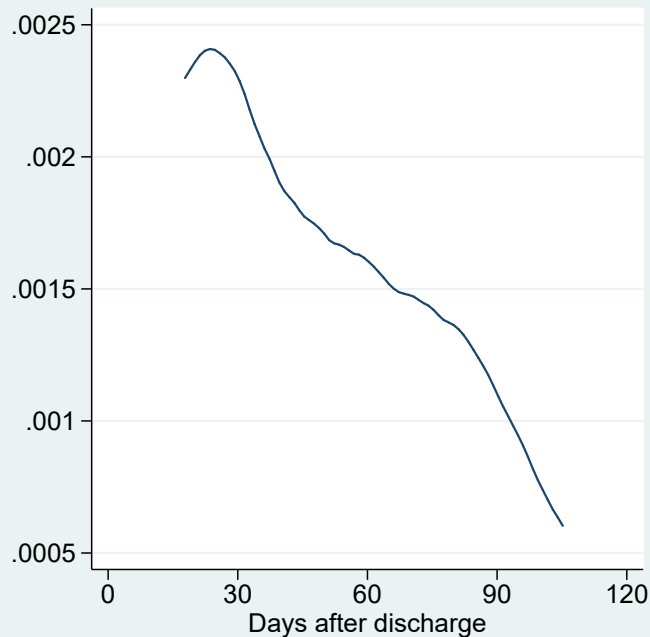

Supplement: S1 Fig — (A) Kaplan-Meier curve; (B) Smoothed hazard estimate (PDF) [file pone.0184402.s003.pdf]
